# Objective Phenotyping of Root System Architecture Using Image Augmentation and Machine Learning in Alfalfa (Medicago sativa L.)

**DOI:** 10.34133/2022/9879610

**Published:** 2022-04-07

**Authors:** Zhanyou Xu, Larry M. York, Anand Seethepalli, Bruna Bucciarelli, Hao Cheng, Deborah A. Samac

**Affiliations:** ^1^USDA-ARS, Plant Science Research Unit, 1991 Upper Buford Circle, St. Paul, MN 55108, USA; ^2^Biosciences Division and Center for Bioenergy Innovation, Oak Ridge National Laboratory, Oak Ridge, TN 37830, USA; ^3^Noble Research Institute, LLC, Ardmore, OK 73401, USA; ^4^Department of Agronomy and Plant Genetics, University of Minnesota, 1991 Upper Buford Circle, St. Paul, MN 55108, USA; ^5^Department of Animal Science, University of California, 2251 Meyer Hall, One Shields Ave., Davis, CA 95616, USA

## Abstract

Active breeding programs specifically for root system architecture (RSA) phenotypes remain rare; however, breeding for branch and taproot types in the perennial crop alfalfa is ongoing. Phenotyping in this and other crops for active RSA breeding has mostly used visual scoring of specific traits or subjective classification into different root types. While image-based methods have been developed, translation to applied breeding is limited. This research is aimed at developing and comparing image-based RSA phenotyping methods using machine and deep learning algorithms for objective classification of 617 root images from mature alfalfa plants collected from the field to support the ongoing breeding efforts. Our results show that unsupervised machine learning tends to incorrectly classify roots into a normal distribution with most lines predicted as the intermediate root type. Encouragingly, random forest and TensorFlow-based neural networks can classify the root types into branch-type, taproot-type, and an intermediate taproot-branch type with 86% accuracy. With image augmentation, the prediction accuracy was improved to 97%. Coupling the predicted root type with its prediction probability will give breeders a confidence level for better decisions to advance the best and exclude the worst lines from their breeding program. This machine and deep learning approach enables accurate classification of the RSA phenotypes for genomic breeding of climate-resilient alfalfa.

## 1. Introduction

Alfalfa (Medicago sativa L., also known as lucerne) is a widely grown perennial forage crop that provides multiple years of soil coverage and accrual of belowground biomass. This plant has a deep root system capable of extracting water and nutrients from as deep as 6 meters ([1]). The extensive crown (consisting of belowground stems) and the root system actively sequester carbon throughout the life of the stand. In addition to carbon sequestration, alfalfa can fix about 200 (4 seasonal harvests) or 650 kg (7 seasonal harvests) of nitrogen ha^−1^ per year through biological nitrogen fixation [2]. However, selection for root system architecture (RSA) traits has lagged behind selection and breeding for aboveground traits due to the high level of morphological plasticity of roots in soil [3–6] and the difficulty of measuring RSA traits [7].

RSA is defined as the spatial distribution of all root parts of a plant over time in a particular growth environment [8]. RSA is controlled by heritable genetics of plants and nonheritable external environmental conditions (soil moisture, temperature, nutrients, and pH) and the microbial communities that impact how a plant detects and responds to its surroundings [9, 10]. Different root characteristics enable plants to respond, adapt, and thrive in different environments, influencing drought tolerance [11], heat tolerance [12], lodging resistance [13], nutrient deficiency [8, 14], and yield [15–17]. RSA determines the extent of the soil volume from which water and nutrients may be acquired. As important as the total volume of soil explored, the distribution of roots in soil is essential for managing the costs of soil foraging by roots [18]. As global climate change occurs, it will be crucial to improve root systems to enhance plant responses to abiotic and biotic stresses. However, using conventional breeding based on phenotypic selection, it is challenging to select breeding lines possessing promising RSA types to adapt to environmental stresses because roots remain hidden underground.

To address the challenge of phenotyping RSA, researchers have explored three strategies [19], including (1) well-controlled laboratory methods [20, 21], moderately controlled greenhouse methods [22, 23], and (3) open field methods [24–26]. The significant challenges are the high labor and time costs in RSA field phenotyping [27, 28] and the generally low correlation between RSA of plants grown in highly controlled growth chambers or greenhouse experiments and plants grown in dynamic environments in the field experiments [29].

To overcome the limitation of the low correlation between field and greenhouse RSA data, many researchers are developing technologies that enable high-throughput phenotyping of RSA traits in the field. However, few low-cost, high-throughput root phenotyping methods are available [30–32]. Shovelomics, or root crown phenotyping, is a widely used method of digging up the root base of plants grown in the field and measuring root characters [28, 33–36]. It is less expensive than some other methods but may provide only limited information on the distal parts of the root system or fine roots, not a picture of the whole root system. Thus, it is still challenging to improve root traits by phenotypic selection during the breeding process.

Results of marker-assisted selection and genomic prediction have higher selection accuracy resulting in higher genetic gains than phenotypic selection. In rice, five QTLs associated with four seedling RSA traits from visual scores and measurements from WinRhizo were identified from both conventional linkage analysis and a machine learning approach via a Bayesian network. Two extreme RSA groups were successfully selected based on the genomic selection rank-sum index [37]. The prediction accuracies of the 13 root architecture traits ranged from the lowest of 0.07 for crossing root to the highest of 0.59 for lateral root tips. Eight QTLs associated with narrow root cone angles of rice RSA mapped with root trait data were stable across glasshouse and three field locations [38]. In canola, 31 QTLs associated with five RSA traits were mapped through genome wide association mapping using visual RSA scoring [33]. Such QTL studies suggest that many traits fundamental to RSA are controlled by numerous small-effect loci [33, 39, 40]. Many QTL studies have relied on visual phenotyping root features or subjective classification of root types. However, these methods are subject to human error and rater bias.

The advent of machine learning (ML) and deep learning (DL) has enabled trait extraction and high throughput phenotyping of many traits. ML has facilitated the development of software tools that automate image processing or data analysis to learn from hidden patterns and classify objects, thus reducing variability in measurements and removing subjectivity and biases [41–43]. Unsupervised learning is a type of machine learning algorithm that learns patterns from unlabeled data. Most unsupervised machine learning is referred to as clustering [44]. For RSA, the expectation is that the machine is forced to classify the roots into distinct clusters based on the internal representation of RSA traits without external interference and human biases. Supervised machine learning is accomplished by various algorithms that can learn the hidden patterns and rules from labeled or tagged training data to predict outcomes for unforeseen data. In supervised learning, the machine is trained using data that is well “labeled” as the ground truth of the data. Kumar et al. (2014) trained their model to recognize and differentiate root tips from 2D images in an automated process [45]. With the power of ML classification and computer vision technology “Zernike Moment Descriptors,” the prediction accuracies were 97% for primary roots and 96% for lateral roots. In pea, by combining random forest and support vector machine models, prediction accuracy for distinguishing cultivars was up to 86% based on the top five RSA traits measured from a greenhouse experiment [46]. In rice, support vector machine (SVM) with 16 image-based RSA traits successfully differentiated 118 genotypes [21].

Most phenotyping of RSA derives from the relatively simple root traits in annual crops, including maize [47, 48], soybean [49–52], rice [21], and Arabidopsis [53], with comparatively little known about the substantially more complicated RSA of perennial plants such as alfalfa (Medicago sativa L.). The roots of alfalfa can grow to depths of 6 meters or more [1] and are important for winter survival [54] and persistence during periods of heat and drought [55, 56]. Previously, branch rooted and taprooted RSA were classified by visual scoring and populations developed for each RSA through two cycles of divergent selection. Heritability of 21 to 48% was attained for branch roots and 11 to 43% for lateral root number [57]. In this study, populations selected for greater root mass had higher forage yields while a deep taproot increased potential access to water resources to improve drought tolerance. Root traits such as taproot diameter or root dry matter may increase winter survival and persistence in alfalfa. The taproot classification implies that the taproot is prominent with few, fine lateral roots, while the branched root system also has a taproot, but it may be less prominent and with more thicker lateral roots. We hypothesize that branched alfalfa roots may be especially important for topsoil foraging [58], while the dominant taproot systems may allow more allocation to deeper root systems [59].

In order to advance root-based breeding in alfalfa, we aimed to develop an imaging protocol based on root crown phenotyping [60] that would allow subsequent automated classification into taproot, branched, and intermediate root types. The objective of this study was to compare unsupervised and supervised machine learning methods as well as deep learning to identify the most promising methods to incorporate into breeding programs for root traits in alfalfa.

## 2. Material and Methods

### 2.1. Plant Materials, Image Capture, and Phenotyping

Five alfalfa populations that were created based on selection for RSA types were used for this study. Starting from a parental population UMN2892, the population UMN3233 was the result of three cycles of phenotypic selection for branch (thin taproot with thicker laterals) roots and UMN3234 the result of three cycles of selection for taprooted (dominant taproot) plants [17, 57]. The selected plants were randomly intermated after each selection cycle, and the resulting progeny was evaluated for the desired root phenotypes. The population UMN4561 (fourth cycle of selection) was developed from UMN 3233 for branch roots using a seedling selection method [61]. Similarly, a fourth cycle of selection was done using the same seedling selection method to produce UMN4563 from UMN3234 for taprooted plants.

The five populations were individually hand seeded into 1.4 m × 0.9 m plots with 28 plants per plot. The plants were equally spaced within the plot using a 13 cm × 13 cm grid. All grid positions were seeded with two to four seeds and thinned to one plant at 21 days after seeding. Each plot was surrounded by a border row of the alfalfa cultivar Agate. Six replicated plots per population were randomly spaced within the field. Planting was done on 1 June 2016 at the University of Minnesota St. Paul Experiment Station (Waukegan fine-silty loam: sandy-skeletal, mixed, superactive, mesic Typic Hapludoll). The plant root system was excavated 20 weeks after planting by digging individual plants to a depth of about 30 cm using a shovel on 12 October 2016. The foliage was removed 4 cm above the crown. Roots were washed to remove soil and stored at 4°C. Root systems were photographed using a Panasonic DMC-FZ30 digital camera held approximately 30 cm above the roots placed on a black background under ambient lighting in a laboratory. The lens was not zoomed so focal length was 35 mm. Root phenotypes were categorized based on visual inspection of the images by an experienced researcher. The branch root (B) phenotype was classified as producing 4-6 thick lateral roots along the taproot at 1 to 2 cm intervals. The taproot (T) phenotype was categorized as having less than four lateral roots emerging from the taproot that were spaced 3 to 4 cm apart. Intermediate phenotypes (TB) had four or more lateral roots spaces more than 2 cm apart and any others neither T nor B types. The total number of individual roots evaluated for each population ranged from 94 to 129, with a total of 617 images. Among the 617 images, 237 or 38.41% of the images are B type, 245 or 39.71% are T type, and 135 or 21.88% are TB type. The detailed information of these 617 images can be found in supplemental Table 1.

### 2.2. Segmentation of Roots and Image Analysis for Feature Extraction

**Figure 1:**
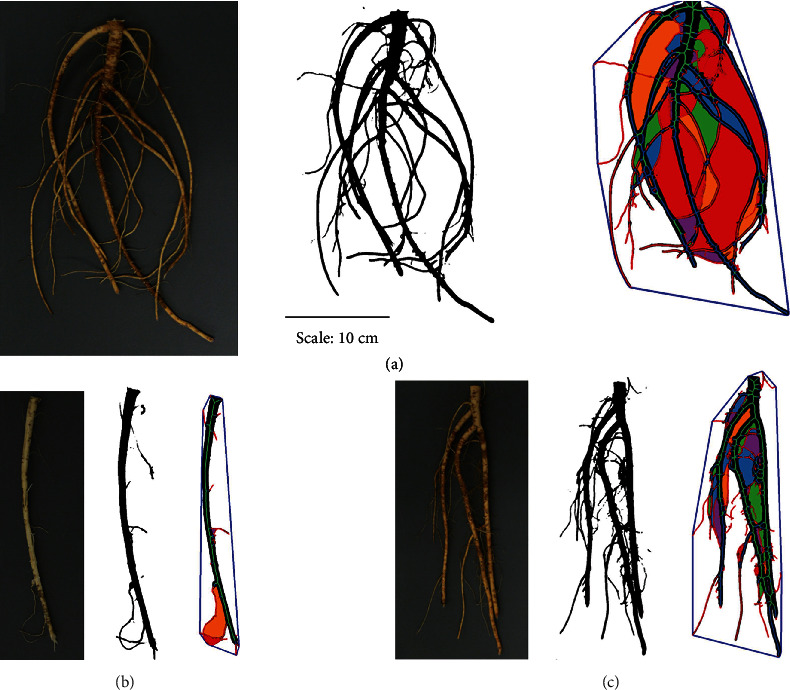
Representative images of (a) branch rooted type, (b) tap rooted type, and (c) intermediate tap-branch root type. For each root type the original raw digital image, segmentation from the background with RootPainter and feature images from RhizoVision Explorer are shown.

The working distance of the camera was not constant during imaging; therefore, before batch image analysis, the pixel width of the circular scale in each image was recorded using ImageJ [62], and the circular tag and ID tag were erased by filling the area with a black background. Since distortion of the root images was minimal because the sample was always in the center of the image where distortion had little effect, no distortion correction to the root images was applied during image processing. To segment the roots from the background, the RootPainter software [63] was used to partially annotate 10 images, focusing on annotating root and background edges as well as the fine lateral roots. The software used built-in neural networks to train the segmentation model over 60 epochs based on these annotations. The resulting network was then used for batch segmentation of all 617 images. The segmented images were further converted to black-on-white binary PNG images using the RootPainter menu item “Convert segmentations for RhizoVision Explorer (Figure 1).”

These binary images were batch analyzed in RhizoVision Explorer v2.0.2 [64] using feature extraction algorithms described and validated by Seethepalli et al. [65]. Analysis settings were “Whole root” mode, no physical unit conversion (left in pixel values), thresholding at 200, root pruning on and set at 2, and with 3 diameter ranges 0-10, 11-20, and 21 and above. The resulting feature data file included measures in pixel values. Using the previously measured circular scales in each image, the number of pixels per mm was computed; then, pixel values were converted to mm, mm^2^, and mm^3^ as appropriate. This resulted in 38 computed root traits including tip number; branch number; branching density; length; area; volume; number of roots; root system width and depth; convex hull area; number and area of holes; angle frequencies; average, median, and maximum diameter; and then the length, surface area, and volume within each diameter range that are described more fully in Seethepalli et al. [65].

### 2.3. Image Augmentation

In order to increase the size of the image set to test improved accuracy through image augmentation, we developed a Python script to automatically create 10 more transformations of each of the 617 segmented images. The functions “getRotationMatrix2D()” and “warpAffine()” from the OpenCV library were used to rotate and scale the images. Rotation was constrained between -20 and 20 degrees, and scaling was limited to between 80% and 120% of the pixel dimensions of the original images. This resulted in realistic images that maintained the overall vertical orientation important for angle measures, similar to simulating arbitrary placement of the root crown by a researcher. For each image, the rotation and scale factors were randomly pulled from the constrained distributions, the original segmented image was transformed, and the resulting image was saved along with a log file of the transformation factors used. This process was repeated 10 times for each original segmented image, resulting in 6,170 augmented images that were processed using RhizoVision Explorer as described above to generate the augmented dataset. To save computation time, we use the augmented images for only deep learning with TensorFlow and RF.

### 2.4. Machine Learning

Unsupervised ML was carried out with *k*-means clustering [66]. We used *k* = 3 for the three groups of RSA types: B, T, and TB. Each of the 38 RSA traits was normalized 0 to 1 by *y*_nor_ = (*y* − *y*_min_)/(*y*_max_ − *y*_min_) because *k*-means clustering is sensitive to the measurement units and numeric values. All the RSA traits were treated with equal weight to calculate the Euclidean distances for classification.

For the centroid-based *k*-means clustering (Model 1), the parameters used for the study were as follows: the number of centers was set as 3 for three clusters of B, T, and TB (centers = 3); the maximum number of iterations to find the best three centroids allowed was set to 100 (iter.max = 100); and the algorithm of Hartigan-Wong was chosen for the *k*-means clustering (algorithm = ^“^Hartigan-Wong”). The *k*-means clustering was implemented with R package “stats” [67]. Partitioning of the data into *k* clusters “around medoids” (PAM; Model 2) is a more robust version of *k*-means unsupervised ML [68]. The clustering function “pam” from R package “cluster” [69] was employed to classify the 617 roots into three root types. PAM clustering is also sensitive to unnormalized numeric values. The same normalized data set was used for classification with the same parameters: *k* = 3 for three clusters of B, T, and TB, and “euclidean” distance was used for the parameter metric (metric = ^“^euclidean^”^).

Two supervised ML algorithms, random forest (RF, Model 3) and naïve Bayes (NB, Model 4), were selected to analyze the root image data for this research. RF trained the prediction model by constructing multiple decision trees with the 38 RSA traits. After constructing the RSA root type trees, the RF method determined the mode of the classes (classification) or mean prediction among all possible decision trees (regression) or the frequency of the correctly predicted RSA type (probability). Random forest classification was conducted with R package “randomForest” [70]. Two parameters, “mtry” (number of variables randomly selected to construct the decision tree) and “ntree” (number of trees to calculate the accuracy and probabilities), were tuned for the RF model. The “mtry” was estimated using formula mtry = floor(sqrt(ncol(root.data.set))), and in our analysis, 6 was the best number of variables for each split. The “ntree” of 500 and 1000 was compared; 500 was selected since it is the default number of trees.

Naive Bayes (NB) is a supervised ML algorithm based on the Bayes Theorem to solve classification problems by following a probabilistic approach [71]. It is based on the assumption that the predictor variables in an ML model are independent. The probability for each of the three RSA types, B, T, and TB, was calculated using the equation of Nwanganga (2020) [72].

NB utilized training data to calculate an observed probability of each of the three RSA types, B, T, and TB, based on the evidence provided by the 38 predicters. NB classification was conducted via R package “e1071” [73]. The parameter of positive double controlling Laplace smoothing was set as 1.

### 2.5. Deep Learning with Neuralnet and TensorFlow

Two DL models, the traditional artificial neural network (ANN) (Model 5) and the TensorFlow-based neural network (Model 6), were used to study the 617 alfalfa root images.

**Figure 2:**
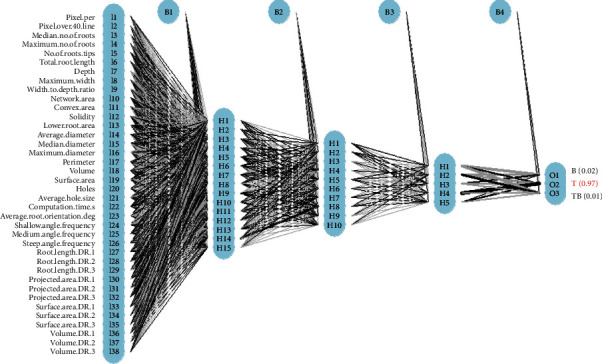
ANN with three hidden layers. The letter “I” stands for the input layer, I1 to I38 are the 38 input predictors: H for hidden layers. H1 to H15 are the 15 neurons of the first hidden layer, 10 neurons in the second hidden layer, and five neurons in the third hidden layer. B1, B2 B3, and B4 for bias to indicate weighted connections between layers. O for output layer, O1 to O3 are the three possible predictions for RSA B, T, and TB root types. The final prediction for the sample in the figure is T, a taproot RSA with 97% probability.

Artificial neural network (ANN) is an ML technique inspired by the biological neural network in the human brain [74]. ANN sends the weight values of each artificial neuron as output to the next layer after processing with inputs from neurons in the previous layer. The backpropagation algorithm is the most widely used training technique to optimize the weights of the neurons. The number of layers, the number of neurons in each hidden layer, and the connection between them were optimized for high prediction accuracy as well as low overfitting. The artificial neural network model forming our system is shown in Figure 2 with five layers: 1 input layer, 3 hidden layers, and 1 output layer. Predicator names and definitions can be found in the supplemental files.

The parameters used for the ANN are ^“^hidden = c (15, 10, 5)^”^ for three hidden layers with 15, 10, and five neurons for three layers. Cross-entropy “ce” is used to calculate the error to evaluate the ANN model (err.fct = ^“^ce^”^). Resilient backpropagation with weight backtracking algorithm, “rpop+”, was selected to optimize the neuron's weight matrix of hidden layers (algorithm = ^“^rprop+^”^). Rectified Linear Unit, “relu,” is an activation function defined as the positive part of its argument, *f*(*x*) = max(0, *x*), where *x* is the input to a neuron is not available for the traditional ANN, so the “logistic” is selected as the activation function to smooth the results of the cross product of the neurons and weights (act.fct = ^“^logistic^”^). The maximum number of steps was 100,000 to train the neural network (stepmax = 100000). Reaching this maximum leads to stopping the neural network's training process without converging to find a reasonable minimum in its loss function. ANN computation was carried out with the R package “neuralnet” [75].

The same neural network structure with the same neurons and layers as in Figure 2 was used to analyze the 617 root image data with TensorFlow [76]. The parameters to run the TensorFlow neural networks were as follows: the activation functions “relu” and “softmax” were selected for the hidden and the output layers, respectively. The loss function “categorical_crossentropy,” the “Adam” optimizer, and quality metrics “accuracy” were selected to train the model. Both ANN and TensorFlow neural networks used 70% and 30% of the data to estimate the prediction accuracy and model stability. The computation of the TensorFlow neural network was carried out using the R package “Keras” Version 2.3.0.0 [77].

### 2.6. Accuracy Metrics

Sensitivity is the estimated frequency of correctly predicted B, T, or TB root types [78]. Sensitivity is calculated as follows:

(1) $$\begin{array}{c} Sensitivity=\frac{\Sigma \;true\;positives\;\left(TP\right)}{\Sigma \;true\;positives\left(TP\right)+\Sigma \;false\;negatives\left(FN\right)}=\frac{\Sigma \;true\;B\;\text{or }T\;\text{or }TB}{\Sigma true\;root\;types+\Sigma \;false\;root\;types}. \end{array}$$

Specificity is the estimated frequency of correct identification as not B or not T or not TB [78]. Specificity is calculated as follows:

(2) $$\begin{array}{c} \text{Specificity}=\frac{\Sigma \text{ true negatives}\left(\text{TN}\right)}{\Sigma \text{ true negatives}\left(\text{TN}\right)+\Sigma \text{ false positives}\left(\text{FP}\right)}=\frac{\Sigma \text{ true not B},\text{T},\text{or TB}}{\Sigma \text{ true not B},\text{T},\text{or TB}+\Sigma \text{ false B},\text{T},\text{or TB}}. \end{array}$$

Precision is used to evaluate the ability to identify the correct root type from among a group consisting of both true root types and falsely identified root types. The higher precision (closer to 1), the lower risk of advancing plants with undesired root types.

(3) $$\begin{array}{c} \text{Precision}=\frac{\Sigma \;true\;positives\;\left(TP\right)}{\Sigma \;true\;positives\left(TP\right)+\Sigma \;false\;positive\left(FP\right)}=\frac{\Sigma \;true\;root\;types}{\Sigma true\;root\;types+\Sigma \;false\;predicted\;root\;types}. \end{array}$$

Prevalence is the proportion of a population who have a specific characteristic, and it is the percentage of positive of all the data and defined as below:

(4) $$\begin{array}{c} \text{Prevalence}=\frac{\Sigma \text{ positives}}{\Sigma \text{ positives}+\Sigma \text{ negatives}}. \end{array}$$

Positive predictive value (PPV) is the percentage of the true positives of all the positive calls.

(5) $$\begin{array}{c} PPV=\frac{\Sigma \;true\;positives\;\left(TP\right)}{\Sigma \;true\;positives\left(TP\right)+\Sigma \;false\;positive\left(FP\right)}=\frac{\Sigma \;true\;root\;types}{\Sigma true\;root\;types+\Sigma \;false\;predicted\;root\;types}. \end{array}$$

Negative predictive value (NPV) is the probability that plants with a negative screening test truly do not have the target root type.

(6) $$\begin{array}{c} \text{NPV}=\frac{\Sigma \text{ true negative }\left(\text{TN}\right)}{\text{TN}+\text{false negative }\left(\text{FN}\right)}. \end{array}$$

Balanced accuracy is the proportion of true positives and true negatives of the three RSA types of B, T, and TB.

(7) $$\begin{array}{c} \text{Balanced accuracy}=\frac{\Sigma \text{ true positive}+\Sigma \text{ true negatives}}{\text{total}}. \end{array}$$

## 3. Results

### 3.1. Unsupervised ML Models Return Similar Results

The two unsupervised ML models generated equivalent classification accuracy of around 70% (Table 1). Both models had higher sensitivity for the B root type and T type than the intermediate TB root type. In Model 2, the sensitivity was 0.738 for the B root type but was only 0.229 for the TB root type. The low sensitivity of TB is consistent with the visual phenotyping in which the TB root types are more difficult and subjective to score. The specificity of Model 1 for TB is larger than that of B and T, but the differences among the three root types are not significant (*p* value > 0.05). The negative predictive values for the B root types are the largest among the seven quality metrics, 0.942 and 0.889 for Models 1 and 2, respectively. Positive predictive values are all close to 0.5, with a mean of 0.5539. High negative and low positive predictive values indicate that predicting the true RSA types will be more challenging than deselecting undesired. The pattern of prevalence from Models 1 and 2 was identical, which the frequency of B (20.7%) < T (31.4%) < TB (47.8%) from Model 1 in the same order for Model 2 with B (25.9%) < T (30.5%) < TB (43.6%). The predicted prevalence pattern showed that unsupervised classification intends to predict the root types as a normal distribution, more for the intermediate TB, and less for B and T root types. Overall, the patterns of the balanced accuracies of the two unsupervised machine learning models were similar. The two unsupervised models grouped more plants into TB than T or B clusters, which was not desired.

### 3.2. Supervised Outperformed Unsupervised Machine Learning

Supervised outperformed unsupervised ML with prediction accuracy around 80% (Table 2), and RF had higher prediction accuracy than the NB model. The RF, the Model 3, had the highest specificity for the B root types among the seven quality metrics, 0.951. TB has the lowest sensitivity among the three root types, as expected in selecting the desired root type, 0.600 and 0.364 for RF and NB models, respectively. Model 3 predicted a much higher frequency (prevalence) of T or B than TB root type. In contrast, the predicted prevalence of root types from the NB for root type B had the lowest frequency of the three root types. Overall, the RF and NB model's balanced accuracies were 0.811 and 0.730, respectively, and RF was significantly better than the naïve Bayes model, with a *p* value of 0.0295.

### 3.3. Deep Learning with Neural Networks Have Potential but with Overfitting Risk

**Figure 3:**
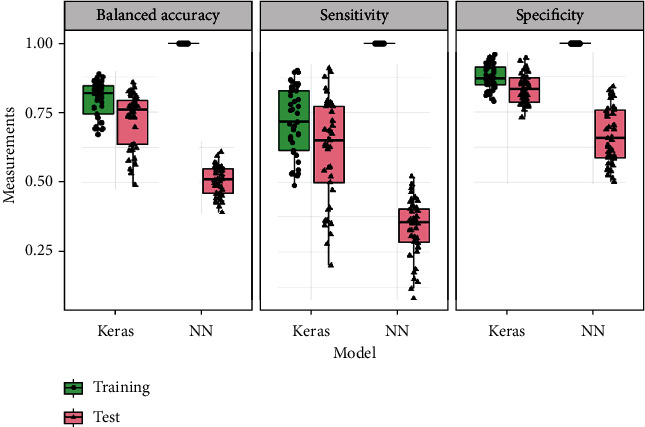
Boxplot of neural network and its overfitting. In each panel, there are four boxplots showing quality metrics from the TensorFlow model implemented with Keras from training data; the TensorFlow model implemented with Keras from test data; the neural network (NN) implemented from the package “neuralnet” from training data, and the neural network implemented from the package “neuralnet” from test data.

DL models showed the advantage of the TensorFlow from the Google Keras application programming interface (API) compared to the traditional neural network implemented from the R package “neuralnet” [79]. The balanced accuracies for B, T, and TB were 0.837, 0.816, and 0.609, respectively (Table 3) from Model 6 Keras/TensorFlow, significantly higher than 0.575, 0.419, and 0.558 from Model 5 neuralnet (*p* value = 0.031). Another noticeable result is severe overfitting of the neural network from the non-tensor-based neuralnet compared with the TensorFlow-based model. The sensitivity, specificity, and balanced accuracy of the training data sets from the three times repeated 5-fold cross-validation were all close to or equal to 100% (Figure 3). Additionally, the sensitivity of the testing data was only about 0.30 from Model 5, and the differences between training and test metrics were highly significant (*p* < 0.01). In contrast, there was no overfitting of the neural network model with the TensorFlow from Keras. The overall mean balanced accuracies from the two DL neural networks were 0.518 and 0.754 for Models 5 and 6, respectively (Table 3), and the TensorFlow neural network outperformed the non-TensorFlow neural network significantly (*p* value < 0.01).

### 3.4. Comparisons among the Unsupervised ML, Supervised ML, and Deep Learning Algorithms

**Figure 4:**
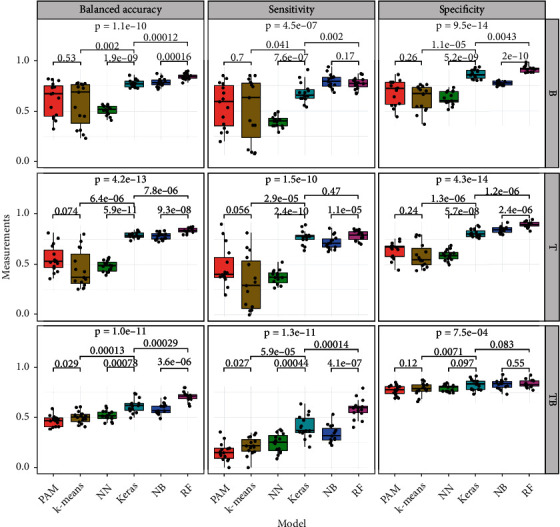
Summary results of three times repeated 5-fold cross-validation from the six models. The values above each boxplot are the *p* values for the pairwise *t*-test, and the value at the top of each boxplot is the *p* value comparing the six models, medoid-PAM (PAM), centroid *k*-means (*k*-means), neuralnet (NN), Keras/TensorFlow (Keras), naïve Bayes (NB), and random forest (RF).

The six models generated a similar pattern for B and T root types from three times repeated 5-fold cross-validation. Decision tree-based random forest had the highest balanced accuracy, 0.843, 0.852, and 0.703 for B, T, and TB root types, respectively (Table 4). In contrast, the unsupervised ML from the partitioning around medoids (PAM) had the lowest balanced accuracy for B root type (0.447) and the largest standard deviations (SD) of 0.180 for T type (Table 4). The considerable variation (Figure 4) of the sensitivity, specificity, and balanced accuracy of the *k*-means and PAM indicates that the unsupervised ML algorithm for the root architecture classification is not stable.

Root type TB had different patterns from that of T and B. Both supervised and unsupervised ML had small standard variations, and all six models for TB root type prediction were stable but small.

All six models except the neuralnet model have the same pattern that the accuracy of B and T root types is larger than that of TB. Neuralnet has the largest balanced accuracy, 0.5146, for the TB of the three root types, which is unexpected. The reason for this exceptional observation may be because of the overfitting of the neuralnet model. Random forest outperformed unsupervised ML models because random forest treats each RSA trait with different weights and some of the decision trees use part of the RSA traits as predictors. In contrast, PAM and *k*-means clustering algorithms use all 38 traits with equal weights for clustering.

### 3.5. Prediction Accuracy Was Improved with Image Augmentation

Prediction accuracies were substantially increased using image augmentation where 6,170 additional images were created from the original 617 by randomly rotating and scaling. The mean balanced accuracies of the RSA types were 0.938 and 0.957 (Table 5), 18.0% and 24.4% higher than those without augmentation for models using TensorFlow-based neuralnet and random forest, respectively. The improved accuracy indicates that DL with TensorFlow had prediction advantages over the ML models when large data sets were used to train the DL model. With improvement from image augmentation, the difference in the prediction accuracy between TensorFlow and RF is not significant, with a *p* value of 0.166. Another noticeable result is the prediction accuracy for the TB root types, the most challenging images to score, is significantly improved (*p* value < 0.01). Overall, image augmentation improves the prediction accuracy for the alfalfa RSA types, and TensorFlow and RF can provide equivalent prediction power and accuracy.

### 3.6. High Prediction Accuracy with High Confidence Level via Prediction Probability

**Figure 5:**
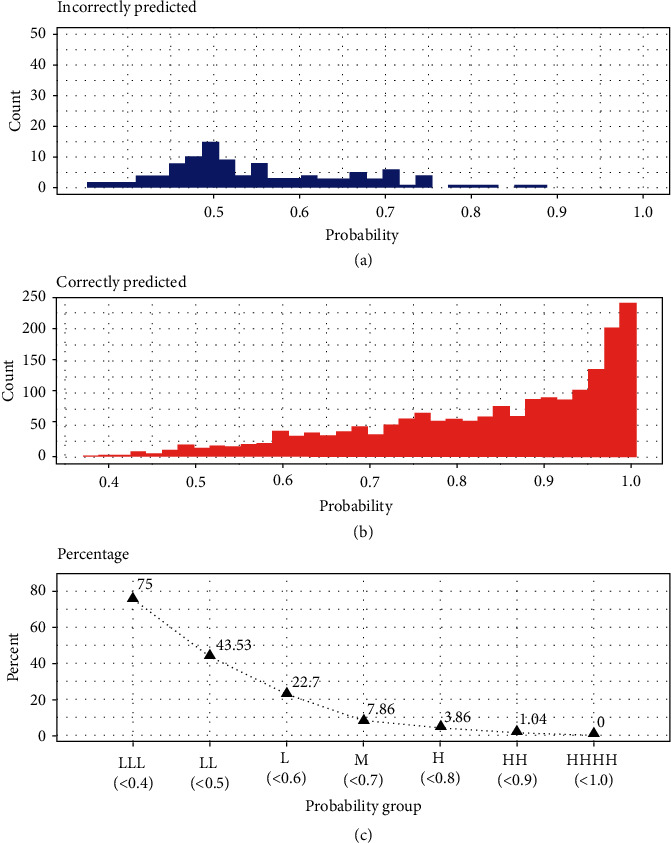
Distribution of the probability for predicting RSA types from RF model.

The default probability threshold for classifying *k* clusters is ≥1/*k*, where *k* is the number of groups and *k* = 3 for this research. Every root will be predicted to be either B or T or TB with three probabilities. If the predicted RSA type with a probability is >1/3, the predicted RSA type will be assigned to that root image. For example, root image name Root002 was predicted with probabilities 0.346, 0.335, and 0.319 for B, T, and TB, respectively, from the RF model (Table 6). Root002 will be assigned to RSA type B since it has the largest probability (0.346) among the three possibilities. This prediction resulted in an incorrectly labeling a TB as B type. The probabilities of the predicted RSA types and predictions were grouped into <0.400 as LLL (L for low confidence level), 0.401 to 0.500 as LL, 0.501 to 0.600 as L, 0.601 to 0.700 as M (M for medium confidence level), 0.701 to 0.800 as H (H for high confidence level), 0.810 to 0.900 as HH, and 0.0901 to 1.00 as HHH. The distributions of the probability from the incorrectly predicted RSA type (Figure 5(a)) and the correct predictions (Figure 5(b)) show that the majority of the incorrectly predicted RSA types have low prediction probability with low confidence levels. The percentage of the incorrectly predicted RSA types among the probabilities less than 0.401 is as high as 75% (Figure 5(c)). The percentage decreased to 3.86% for RSA types with the predicted probabilities between 70 and 80% and further decreased close to 0% for the RSA types with prediction probabilities between 90 and 100%. Thus, by retaining only those plants with roots predicted to be a particular type with a probability greater than 90%, breeders can select the desired RSA types with nearly 100% accuracy.

## 4. Discussion

### 4.1. Selection of the Best Model for Alfalfa RSA Classification

Overall, supervised models outperformed unsupervised ML models for RSA classification in alfalfa. These results may be because the supervised ML can learn the hidden pattern and rules of the RSA root types from the human-created labels and that the data from the 617 root images is highly skewed to both left for B and right end for T root types. The 617 plants are from four cycles of divergent recurrent selection that selected the plants with extreme T or B and discarded the plants with TB roots. The frequencies of the T and B are much higher than that of TB root types due to the breeder's selection scheme. In terms of predicted prevalence, the deep neural network outperforms both unsupervised and supervised ML. The two deep learning models have the most accurate prediction (23.5% of TB type in Table 3). In terms of balanced accuracy, RF was the best of the six models in identifying T and B traits, and TensorFlow from Keras was the second best but the differences were not significant (*p* value > 0.05). TensorFlow did not outperform RF, probably because of the small number of images used for this study. With more images used for the model training, DL can be superior for RSA prediction for root breeding. With small number of images available for an individual breeding program, RF should be preferred due to its computational simplicity and speed. In our study, image augmentation significantly improved prediction accuracy, highlighting the potential of this approach, also called few-shot learning, for plant phenotyping.

### 4.2. Weight of RSA Trait Matters for Supervised and Unsupervised ML

Different traits contribute to the prediction accuracy of ML with varying levels of importance, which may be the reason for low prediction accuracy of unsupervised ML models. The mathematical calculation of the unsupervised *k*-means and PAM models weigh all the 38 RSA traits equally. In contrast, supervised ML assigned different weights for the 38 traits. The importance of the 38 predictors from the RF model ranged from 6 Gini index reduction for the “number of holes” to 25 Gini index reduction for the “lower root area” trait in the RSA structure (supplemental Figure 1). One of the main advantages of DL is optimizing the weights for the original 38 traits at the input layer and the neurons in the hidden layers to increase prediction accuracy. Our observations from this RSA classification study are consistent with observations using pea plants where selecting “top important” root traits provided a significantly improved classification compared to using all available traits or randomly selected trait sets [46]. Another reason for the low classification accuracy of the unsupervised ML is the collinearity of the 38 traits. The correlation coefficients of four traits are highly correlated with a value of 0.9999. ML can select significant predictors and exclude collinear variables, whereas unsupervised ML uses all the predictors with the same weights. Weights of RSA traits affected ML models in numerous other studies [80–83]. In this study, we segmented root crowns and used RhizoVision Explorer to extract root traits for use in these models. More recently, direct classification of images without feature extraction has become more popular in computer vision. This is an exciting opportunity to explore; however, as the extracted root traits such as root length, angles, diameters, and total size are important to consider themselves, we believe the proposed pipeline considered here is relevant and useful for breeding already.

This research focused on image classification for the RSA types instead of treating RSA traits as the continuous numeric measurements for ML regression. ML regression approach could be used to predict the numeric values to cross-validate the classification results if RSA traits were collected as numeric variables. However, we are limited to this approach because the historical visual approach used was only based on categorical classification. But it is possible to use score values for identifying extremes to converge on the same roots and the probabilistic method we used here.

We are optimistic about the results and future application of the approach developed in this research for RSA classification. With 97% prediction accuracy, we showed that automated image analysis and ML could be used for perennial alfalfa RSA prediction with high confidence. One caveat is that alfalfa is a perennial crop that can be cultivated for four to seven years with one planting. The RSA is continually growing and changing based on internal genetics, external environments, and surrounding microbes across the cultivation years. The root samples used in this research are one-time sampling from the field. The prediction accuracy from this research may change due to the stage and time the root samples are collected. More investigations are needed to validate this approach with multiple sampling dates, especially field sampling across years. The imaging method could be improved using the RhizoVision Crown platform that combines a monochrome camera and a backlight to capture root crown silhouettes that facilitates downstream image analysis [84]. In the future, we envision the possibility of using this imaging platform combined with imaging software that contains the trait extraction algorithms of RhizoVision Explorer along with the predicition models in order to classify root types as they are imaged in the field. Stem cuttings could be retrieved from the target plants for vegetative propagation. This automated, unbiased root classification system would be an unprecedented opportunity to breed for root traits in alfalfa to support sustainable agroecosytems.

## Figures and Tables

**Table 1:** Summary of the quality metrics of the unsupervised machine learning models centroid *k*-means and medoid-PAM to distinguish root system architecture in alfalfa. B is for branch rooted type, T is for tap rooted, and TB is intermediate. *μ* is the mean for each metric.

Metric	Model 1: centroid *k*-means				Model 2: medoid-PAM			
	B	T	TB	*μ*	B	T	TB	*μ*
Sensitivity	0.828	0.763	0.305	0.632	0.738	0.658	0.229	0.541
Specificity	0.732	0.771	0.860	0.788	0.740	0.805	0.786	0.777
Positive predictive value	0.447	0.604	0.667	0.573	0.498	0.722	0.319	0.513
Negative predictive value	0.942	0.876	0.575	0.798	0.889	0.753	0.699	0.780
Precision	0.447	0.604	0.667	0.573	0.498	0.722	0.319	0.513
Prevalence	0.207	0.314	0.478	0.333	0.259	0.436	0.305	0.333
Balanced accuracy	0.780	0.767	0.583	0.710	0.739	0.731	0.507	0.659

**Table 2:** Summary of the quality metrics of the supervised machine learning models RF and NB to distinguish root system architecture in alfalfa. B is for branch rooted type, T is for tap rooted type, and TB is intermediate. *μ* is the mean for each metric.

Metric	Model 3: random forest				Model 4: naïve Bayes			
	B	T	TB	*μ*	B	T	TB	*μ*
Sensitivity	0.779	0.803	0.600	0.727	0.817	0.727	0.364	0.636
Specificity	0.951	0.910	0.821	0.894	0.779	0.850	0.843	0.824
Positive predictive value	0.931	0.859	0.341	0.710	0.582	0.784	0.496	0.621
Negative predictive value	0.835	0.871	0.930	0.879	0.918	0.806	0.757	0.827
Precision	0.931	0.859	0.341	0.710	0.582	0.784	0.496	0.621
Prevalence	0.460	0.406	0.134	0.333	0.274	0.428	0.298	0.333
Balanced accuracy	0.865	0.856	0.711	0.811	0.798	0.789	0.604	0.730

**Table 3:** Summary of the quality metrics of the deep learning neural network models neuralnet and Keras/TensorFlow to distinguish root system architecture in alfalfa. B is for branch rooted type, T is for tap rooted type, and TB is for an intermediate type. *μ* is the mean for each metric.

Metric	Model 5: neuralnet				Model 6: Keras/TensorFlow			
	B	T	TB	*μ*	B	T	TB	*μ*
Sensitivity	0.194	0.296	0.591	0.36	0.917	0.761	0.295	0.658
Specificity	0.957	0.543	0.524	0.675	0.757	0.871	0.923	0.85
Positive predictive value	0.737	0.284	0.277	0.432	0.702	0.783	0.542	0.675
Negative predictive value	0.655	0.558	0.806	0.673	0.935	0.856	0.81	0.867
Precision	0.737	0.284	0.277	0.432	0.702	0.783	0.542	0.675
Prevalence	0.385	0.38	0.235	0.333	0.385	0.38	0.235	0.333
Balanced accuracy	0.575	0.419	0.558	0.518	0.837	0.816	0.609	0.754

**Table 4:** Mean and standard deviation (SD) of balanced accuracy from the three times repeated 5-fold cross-validation for the four machine learning and two deep learning models.

Machine learning type	Model name	Branch (B)		Taproot (T)		Between T and B (TB)	
		Mean	SD	Mean	SD	Mean	SD
Unsupervised machine learning	*k*-Means	0.673	0.135	0.598	0.138	0.591	0.122
	PAM	0.447	0.164	0.525	0.180	0.452	0.169
Supervised machine learning	Naïve Bayes	0.787	0.041	0.779	0.036	0.581	0.054
	Random forest	0.843	0.029	0.852	0.031	0.703	0.071
Deep learning	Neuralnet	0.501	0.056	0.487	0.049	0.508	0.053
	TensorFlow	0.789	0.029	0.791	0.037	0.623	0.068

**Table 5:** Summary of the quality metrics from deep learning models using the neuralnet and TensorFlow with image augmentation to distinguish RSA in alfalfa. B is for branch rooted type, T is for tap rooted type, and TB is intermediate. *μ* is the mean for each metric.

Metrics	TensorFlow				Random forest			
	B	T	TB	*μ*	B	T	TB	*μ*
Sensitivity	0.969	0.931	0.841	0.914	0.963	0.946	0.912	0.94
Specificity	0.952	0.968	0.969	0.963	0.981	0.970	0.969	0.973
Positive predictive value	0.930	0.950	0.878	0.919	0.971	0.953	0.884	0.936
Negative predictive value	0.979	0.956	0.958	0.964	0.975	0.965	0.977	0.972
Precision	0.930	0.950	0.878	0.919	0.971	0.953	0.884	0.936
Prevalence	0.398	0.392	0.210	0.333	0.402	0.395	0.203	0.333
Balanced accuracy	0.960	0.950	0.905	0.938	0.972	0.958	0.941	0.957

**Table 6:** Prediction of RSA types, probabilities, and confidence levels from the RF model. Predictions with larger probabilities have higher confidence level than those with lower probabilities.

Image name	RSA type	Prob for B	Prob for T	Prob for TB	Max prob	Predicted RSA type	Correct (Y/N)	Conf level
Root001	TB	0.340	0.325	0.335	0.340	B	No	LLL
Root002	TB	0.346	0.335	0.319	0.346	B	No	LLL
Root003	B	0.328	0.355	0.317	0.355	T	No	LLL
Root004	B	0.351	0.288	0.361	0.361	TB	No	LLL
Root005	B	0.362	0.282	0.356	0.362	B	Yes	LLL
Root006	TB	0.296	0.362	0.342	0.362	T	No	LLL
Root007	T	0.368	0.283	0.349	0.367	B	No	LLL
Root008	B	0.502	0.234	0.263	0.502	B	Yes	LL
Root009	B	0.503	0.237	0.260	0.503	B	Yes	LL
Root010	TB	0.508	0.286	0.206	0.508	B	No	LL
Root011	T	0.175	0.317	0.508	0.508	TB	No	LL
Root012	T	0.229	0.508	0.263	0.508	T	Yes	LL
Root013	T	0.130	0.362	0.509	0.508	TB	No	LL
Root014	B	0.820	0.041	0.139	0.820	B	Yes	HH
Root015	TB	0.820	0.071	0.109	0.820	B	No	HH
Root016	T	0.005	0.990	0.005	0.990	T	Yes	HHH
Root017	B	0.994	0.000	0.006	0.994	B	Yes	HHH

## Data Availability

The original images with tags removed and segmented images from RootPainter for data analysis are available on Zenodo doi:10.5281/zenodo.5879778 [85].

## Supplementary Materials

Supplementary Materials There is two supplemental material associated with this manuscript. One is a supplementary table, and the other one is a figure. The table summarizes the number of root types from different populations and their frequency in percentage. The first number in each cell is the number of root types, and the number inside parenthesis is the percent (%) of the root type. The figure shows the trait importances associated with the RSA. The *x*-axis is the mean decrease in the Gini index (MeanDecreaseGini) and the total decrease in node impurity of a trait. *y*-axis is the 38 traits.
