# Supplementary material for: Objective Phenotyping of Root System Architecture Using Image Augmentation and Machine Learning in Alfalfa (Medicago sativa L.)
Source: Plant Phenomics. 2022 Apr 7;2022:9879610. doi: 10.34133/2022/9879610 (PMC9012978; doi:10.34133/2022/9879610)
Supplement: Supplementary Materials — There is two supplemental material associated with this manuscript. One is a supplementary table, and the other one is a figure. The table summarizes the number of root types from different populations and their frequency in percentage. The first number in each cell is the number of root types, and the number inside parenthesis is the percent (%) of the root type. The figure shows the trait importances associated with the RSA. The x-axis is the mean decrease in the Gini index (MeanDecreaseGini) and the total decrease in node impurity of a trait. y-axis is the 38 traits. [file 9879610.f1.docx]

**Supplemental Figures and Table for**

**Objective phenotyping of root system architecture using image augmentation and machine learning in alfalfa (*Medicago sativa* L.)**

Zhanyou Xu^1*^, Larry M. York^2*^, Anand Seethepalli^3^, Bruna Bucciarelli^4^, Hao Cheng^5^, Deborah A. Samac^1^

^1^ USDA-ARS, Plant Science Research Unit, 1991 Upper Buford Circle, St. Paul, MN 55108

^2^Biosciences Division and Center for Bioenergy Innovation, Oak Ridge National Laboratory, Oak Ridge, TN 37830

^3^Noble Research Institute, LLC, Ardmore, OK 73401

**^4^** Department of Agronomy and Plant Genetics, University of Minnesota, 1991 Upper Buford Circle, St. Paul, MN 55108

**^5^** Department of Animal Science, University of California, 2251 Meyer Hall, One Shields Ave., Davis, CA 95616

*corresponding author

Supplemental Table 1. Summary of the number of root types from different populations and their frequency in percentage. The first number in each cell is the number of root type, and the number inside parenthesis is the percent % of the root type.

| Population | B | T | TB | total |
| --- | --- | --- | --- | --- |
| 2892 | 50 (48.08) | 37 (35.58) | 17 (16.35) | 104 (100) |
| 3233 | 49 (37.4) | 50 (38.17) | 32 (24.43) | 131 (100) |
| 3234 | 50 (39.06) | 48 (37.5) | 30 (23.44) | 128 (100) |
| 4561 | 59 (44.03) | 43 (32.09) | 32 (23.88) | 134 (100) |
| 4563 | 29 (24.17) | 67 (55.83) | 24 (20.00) | 120 (100) |
| total | 237 (38.41) | 245 (39.71) | 135 (21.88) | 617 (100) |


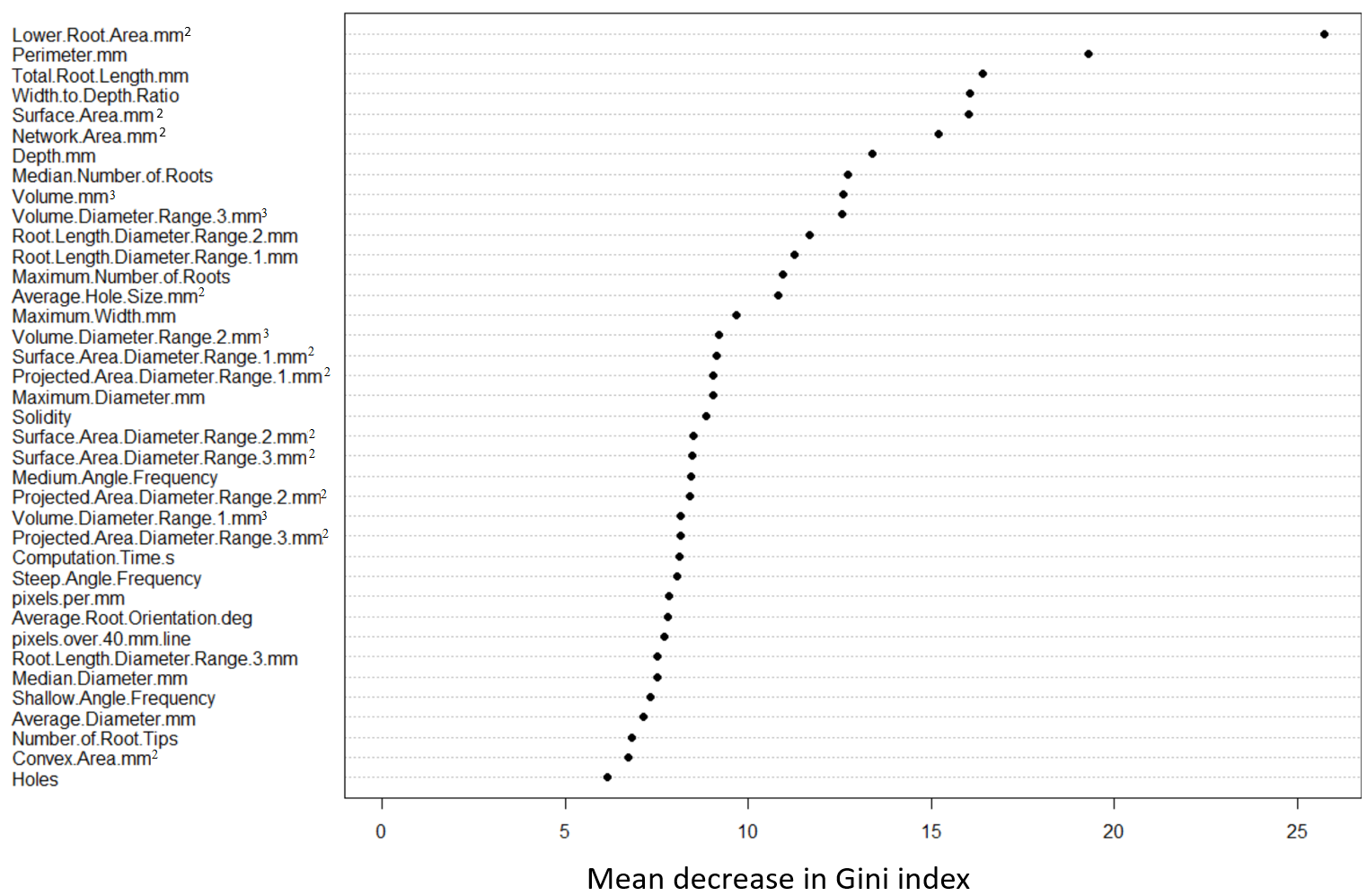


Supplemental Fig. 1. Trait importances of the 38 traits. The X-axis is the mean decrease in Gini index (MeanDecreaseGini), the total decrease in node impurity of a trait. Y-axis is the 38 traits.
